# Supplementary material for: MetaRibo-Seq measures translation in microbiomes
Source: Nat Commun. 2020 Jun 29;11:3268. doi: 10.1038/s41467-020-17081-z (PMC7324362; doi:10.1038/s41467-020-17081-z)
Supplement: Supplementary file 10 — Supplementary Data 7 [file 41467_2020_17081_MOESM10_ESM.zip › File2/Confidence_High_Taxonomy/2348_out.krona.html]

Javascript must be enabled to view this page.

members
magnitude
magnitudeUnassigned
count
unassigned
taxon
rank

2348\_out

36

36
2
superkingdom

36
1239
phylum

36
186801
class

order
186802
36

541000
family
36

36
genus
216851

36

SRS013476\_contig\_number\_38216SRS013638\_contig\_number\_16432SRS013940\_contig\_number\_21545SRS013951\_contig\_number\_36950SRS013965\_contig\_number\_23874SRS014613\_contig\_number\_contig-100\_13241.13241SRS014855\_contig\_number\_6937SRS016018\_contig\_number\_contig-100\_7522.66704SRS016954\_contig\_number\_21075SRS019161\_contig\_number\_40694SRS019787\_contig\_number\_11047SRS024132\_contig\_number\_contig-100\_862.292061SRS024625\_contig\_number\_contig-100\_1352.59277SRS042628\_contig\_number\_22438SRS045826\_contig\_number\_5917SRS053398\_contig\_number\_contig-100\_6403.110680SRS063985\_contig\_number\_11723SRS064232\_contig\_number\_contig-100\_3147.30745SRS075078\_contig\_number\_30241SRS076976\_contig\_number\_14740SRS077231\_contig\_number\_23283SRS078419\_contig\_number\_contig-100\_2014.218211SRS097958\_contig\_number\_contig-100\_763.145959SRS101376\_contig\_number\_contig-100\_10904.115138SRS1041133\_contig\_number\_12450SRS1041134\_contig\_number\_contig-100\_17943.17944SRS142503\_contig\_number\_contig-100\_18127.189588SRS142781\_contig\_number\_contig-100\_25198.68256SRS147139\_contig\_number\_45066SRS147377\_contig\_number\_11993SRS147653\_contig\_number\_contig-100\_249.55580SRS148424\_contig\_number\_27476SRS149879\_contig\_number\_11767SRS893300\_contig\_number\_contig-100\_34237.34238SRS893373\_contig\_number\_21046SRS893383\_contig\_number\_22865
species
853
